# Randomized, double-blind trial of F14512, a polyamine-vectorized anticancer drug, compared with etoposide phosphate, in dogs with naturally occurring lymphoma

**DOI:** 10.18632/oncotarget.27461

**Published:** 2020-02-18

**Authors:** Pierre Boyé, Franck Floch, François Serres, Zacharie Segaoula, Juliette Hordeaux, Quentin Pascal, Virginie Coste, Sandy Courapied, Emmanuel Bouchaert, Agata Rybicka, Claire Mazuy, Laurent Marescaux, Kévyn Geeraert, Corinne Fournel-Fleury, Alain Duhamel, François Machuron, Pierre Ferré, Aurélie Pétain, Nicolas Guilbaud, Dominique Tierny, Bruno Gomes

**Affiliations:** ^1^ OCR (Oncovet-Clinical-Research), Loos, France; ^2^ Oncovet, Villeneuve d’Ascq, France; ^3^ Université de Lille, JPARC - Centre de Recherche Jean-Pierre Aubert, Neurosciences et Cancer, Lille, France; ^4^ Laboratoire Fleury-Fournel ALVEDIA, Limonest, France; ^5^ Université Lille, Santé Publique: Epidémiologie et Qualité des Soins, Lille, France; ^6^ Institut de Recherche Pierre Fabre, Toulouse, France; ^7^ Current address: Department of Small Animal Teaching Hospital, The Royal (Dick) School of Veterinary Studies and The Roslin Institute, University of Edinburgh, UK; ^8^ Current address: Hoffmann-La Roche, Switzerland

**Keywords:** etoposide phosphate, F14512, non-Hodgkin lymphoma, pet dog model, P-glycoprotein

## Abstract

**Purpose:** F14512 is an epipodophyllotoxin derivative from etoposide, combined with a spermine moiety introduced as a cell delivery vector. The objective of this study was to compare the safety and antitumor activity of F14512 and etoposide phosphate in dogs with spontaneous non-Hodgkin lymphoma (NHL) and to investigate the potential benefit of F14512 in P-glycoprotein (Pgp) overexpressing lymphomas.

**Experimental Design:** Forty-eight client-owned dogs with intermediate to high-grade NHL were enrolled into a randomized, double-blind trial of F14512 *versus* etoposide phosphate. Endpoints included safety and therapeutic efficacy.

**Results:** Twenty-five dogs were randomized to receive F14512 and 23 dogs to receive etoposide phosphate. All adverse events (AEs) were reversible, and no treatment-related death was reported. Hematologic AEs were more severe with F14512 and gastrointestinal AEs were more frequent with etoposide phosphate. F14512 exhibited similar response rate and progression-free survival (PFS) as etoposide phosphate in the global treated population. Subgroup analysis of dogs with Pgp-overexpressing NHL showed a significant improvement in PFS in dogs treated with F14512 compared with etoposide phosphate.

**Conclusion:** F14512 showed strong therapeutic efficacy against spontaneous NHL and exhibited a clinical benefice in Pgp-overexpressing lymphoma superior to etoposide phosphate. The results clearly justify the evaluation of F14512 in human clinical trials.

## INTRODUCTION

Comparative oncology has shown that naturally occurring canine cancers are of valuable and translatable interest for the understanding of human cancer biology and the characterization of new therapies [1–3]. Dogs develop a broad spectrum of spontaneous occurring cancers that share strong similarities with human cancers, offering a singular opportunity to answer key questions (efficacy, safety, pharmacokinetic/pharmacodynamic, biomarkers) and guiding the cancer drug development path in a manner not possible using more conventional models [1–3].

Non-Hodgkin lymphomas (NHL) are among the most common hematopoietic cancers in both human and dog populations. Canine NHL share many biological and therapeutic similarities with their human counterparts, including clinical presentation, biological behavior, and therapeutic responses [4–6]. These similarities support the use of the canine model as a comparative, relevant large animal model to study new therapies for both human and canine benefit.

Multidrug resistance (MDR) is one major cause of treatment failure in most malignant hematopoietic cancers reported in dogs and humans, associated with high rates of mortality [7–10]. The most common mechanism of MDR is the overexpression of ATP binding cassette (ABC) transporters, which actively pump numerous chemotherapeutic drugs outside the cancer cells, attenuating drug efficacy and resulting in resistance to treatment [10–12]. The ABC transporter subfamily B member 1 (ABCB1/MDR1, P-glycoprotein, Pgp) is one of the most important transporters to confer MDR to cancer cells [13–16].

F14512 is an epipodophyllotoxin (etoposide) core combined with a spermine chain able to specifically target cancer cells with an active polyamine transport system (PTS) [17, 18]. The positively charged spermine tail contributes to (i) favoring the selective uptake of the drug by tumor cells via the PTS, (ii) increasing DNA binding to reinforce topoisomerase II inhibition, (iii) enhancing the water solubility of the drug [17–20]. The antiproliferative activity of F14512 has been demonstrated to be superior to etoposide in numerous human cancer cell lines such as breast cancer, non-small cell lung cancer, leukemia, melanoma, ovarian cancer and carcinomas [17, 21–24]. In a vinorelbine-resistant P388 mouse leukemia cell line model overexpressing high level of functional P-glycoproteins, F14512 displayed a strong antileukemic activity and the antitumor activity of F14512 was not impacted by the MDR status of cancer cells [Annereau JP, Brel V, Riquet W, Créancier L, Vandenberghe I, Fournier E, Robichon C, Stennevin A, Offrete V, Lacastaigneratte L, Gomes B, Kruczynski A, Bailly C, *et al*. Abstract 988: F14512, a novel vectorized topoiserase II inhibitor, bypasses MDR1 mediated resistance. Abstract presented at the 104th Annual Meeting of the American Association for Cancer Research, Washington, DC, 6 to 10 April 2013]. These characteristics augured well for further development of the drug in malignant hematopoietic tumor patients.

On the basis of these preclinical data, we initially evaluated the safety, pharmacokinetic and pharmacodynamic profiles of F14512 in dogs with spontaneous NHL in a traditional 3+3 phase I dose-escalation trial [25]. F14512 was well tolerated in dogs, and F14512 demonstrated a strong antitumor activity on canine NHL. The safety and tolerability of intravenous etoposide phosphate were also investigated to determine the maximum tolerated dose in dogs [26]. These results provided the rationale for the phase II, randomized, double-blind trial in dogs. The objective of this study was to compare the safety and antitumor activity of F14512 and etoposide phosphate in dogs with spontaneous NHL and to investigate the potential benefit of F14512 in P-glycoprotein overexpressing lymphomas, with the primary aim of showing that effectively designed comparative oncology studies would offer bidirectional benefit to both pet animals and humans with cancer.

## RESULTS

### Epidemiological and clinical characteristics

**Figure 1:**
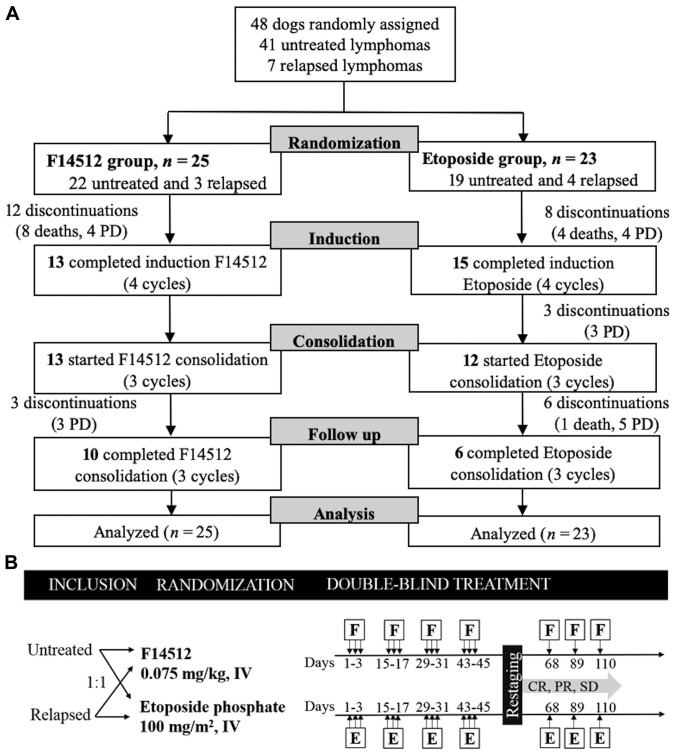
Trial design. (**A**) Flow diagram for the trial. Enrollment of 48 dogs randomized to receive F14512 (*n* = 25) or etoposide phosphate (*n* = 23). (**B**) Schematics of study design. Black arrows indicate time of treatment administration. CR: complete response, E: etoposide phosphate, F: F14512, IV: intravenous, PD: progressive disease, PR: partial response, SD: stable disease.

Between December 03, 2014 and August 30, 2016, 48 dogs with naturally occurring intermediate to high-grade NHL were enrolled in this randomized double-blind clinical trial. Forty-one (41/48, 85%) dogs had no prior treatment, and 7 (7/48, 15%) dogs were enrolled after a relapse following a CHOP-based chemotherapy protocol. Twenty-two (22/48, 46%) dogs with untreated NHL were randomized to receive F14512 and 19 (19/48, 40%) to receive etoposide phosphate. Three (3/48, 6%) dogs with relapsed NHL were randomized to receive F14512 and 4 (4/48, 8%) to receive etoposide phosphate. The last day of follow-up was January 30, 2017. All dogs in both treatment groups received the same 8-week induction chemotherapy protocol, consisting of a 3-hour, once daily intravenous (IV) injection on 3 consecutive days, and every 2 weeks for 4 cycles. After completion of the 4 cycles, dogs who experienced a clinical benefit (complete or partial response or stable disease) received 3 additional consolidation cycles with one intravenous injection of the same drug every 3 weeks (day 68, 89, and 110) (Figure 1). The main clinical characteristics were balanced between the two treatment groups and are summarized in Table 1. Dogs treated with F14512 were statistically younger than dogs treated with etoposide phosphate (mean ± SD: 6.6 ± 2.5 years *versus* 8.5 ± 2.7 years, *P* = 0.016).

**Table 1:** Epidemiological and clinical characteristics of dogs randomized to receive F14512 (*n* = 25) or etoposide phosphate (*n* = 23)

Epidemiological characteristics	Total	F14512	Etoposide phosphate	*P* value
**Number of dogs (%)**	**48 (100%)**	25 (100%)	23 (100%)	
**Prior treatment, no. (%)**				0.696^a^
Untreated	**41 (85%)**	22 (88%)	19 (83%)	
Prior chemotherapy	**7 (15%)**	3 (12%)	4 (17%)	
**Sex, no. (%)**				0.087^b^
Male	**27 (56%)**	17 (68%)	10 (43%)	
Female	**21 (44%)**	8 (32%)	13 (57%)	
**Age, years, mean ± SD (range)**	**7.5** ± **2.7 (3–14)**	6.6 ± 2.5 (3–12)	8.5 ± 2.7 (5–14)	0.016^c^
**Body weight, kg, mean ± SD (range)**	**28.5** ± **11.7 (8.0–51.4)**	31.5 ± 10.2 (8.0–48.8)	25.3 ± 12.6 (8.6–51.4)	0.067^c^
**Clinical stage at inclusion, no. (%)**				0.482^a^
Stage II	**2 (4%)**	—	2 (9%)	
Stage III	**8 (17%)**	4 (16%)	4 (17%)	
Stage IV	**29 (60%)**	17 (68%)	12 (52%)	
Stage V	**9 (19%)**	4 (16%)	5 (22%)	
**Clinical substage (systemic signs), no. (%)**				0.398^b^
Substage a (absence)	**26 (54%)**	15 (60%)	11 (48%)	
Substage b (presence)	**22 (46%)**	10 (40%)	12 (52%)	
**Immunophenotype, no. (%)**				0.88^a^
B-cell	**37 (77%)**	20 (80%)	17 (74%)	
T-cell	**7 (15%)**	3 (12%)	4 (17%)	
Unclassified	**4 (8%)**	2 (8%)	2 (9%)	
**Cytomorphological Subtype, no. (%)**				1.0^a^
- B-cell lymphoma:				
DLBCL	**33 (69%)**	17 (68%)	16 (70%)	
Transformed marginal zone lymphoma	**2 (4%)**	1 (4%)	1 (4%)	
Burkitt-like		2 (8%)	—	
- T-cell lymphoma:	**2 (4%)**			
T-cell Pleomorphic	**1 (2%)**	—	1 (4%)	
Large granular T-cell	**1 (2%)**	1 (4%)	—	
Transformed small clear cell, T-zone	**1 (2%)**	—	1 (4%)	
High-grade, small to medium cells, unspecified	**3 (6%)**	2 (8%)	1 (4%)	
- Unclassified	**5 (11%)**	2 (8%)	3 (13%)	
**Pgp expression at inclusion, no. (%)**				1.0^a^
Pgp- (neg and low)	**16 (33%)**	8 (32%)	8 (35%)	
Pgp+ (mid and high)	**28 (59%)**	15 (60%)	13 (57%)	
Unclassified	**4 (8%)**	2 (8%)	2 (8%)	

DLBCL: Diffuse large B-cell lymphoma, Pgp: P-glycoprotein. (a) Fisher’s exact test; (b) Chi-square test; (c) *t*-test.

### P-glycoprotein (Pgp) expression in tumor cells

**Figure 2:**
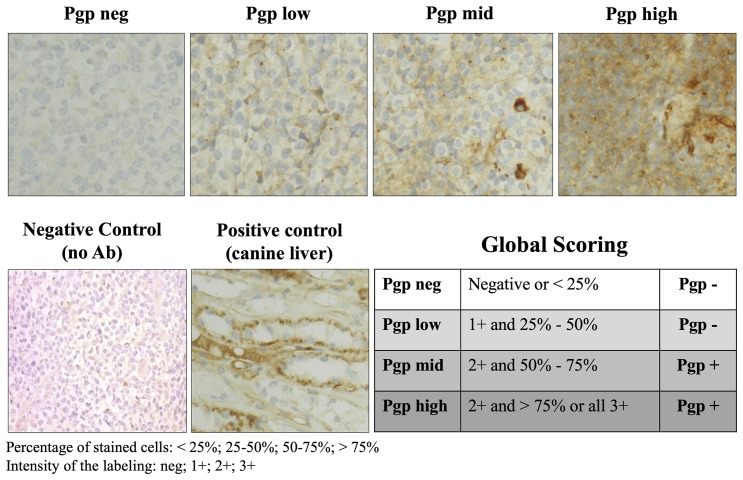
P-glycoprotein expression evaluated by immunohistochemistry in lymph node tumor cells. Microscopic photographs of lymph node histology stained with a primary mouse anti-human P-glycoprotein clone C494 antibody (ALX-801–003-C100, lab Enzo life sciences). Original magnification ×40. Normal canine liver was used as a positive control for P-glycoprotein analysis. Non-immune serum substituted to C494 on lymph node tissues was used as a negative control. Immunohistochemical score method was then evaluated by a scoring system adapted from Lee and colleagues as follows: negative (neg, less than 25% stained cells), low (more than 25% but less than 50% of the tumor cells are stained with a light staining), moderate (mid, more than 50% but less than 75% of the tumor cells are stained with moderate intensity), high (more than 75% are stained with a moderate to high intensity). Negative and Low Pgp expression was included in the Pgp- group; Mid and High expression was included in the Pgp+ group. Ab: antibody; Pgp: P-glycoprotein.

P-glycoprotein expression was evaluated in all dog tumor cells by immunohistochemistry (IHC) using the monoclonal antibody C494 adapted from Lee and colleagues [13] (Figure 2). Twenty-eight (28/48, 59%) dogs were classified with Pgp-positive tumors (Pgp+, high and moderate expression) and 16 (16/48, 33%) dogs were Pgp-negative (Pgp-, negative and low expression). In the group of dogs treated with F14512, 15 (15/25, 60%) dogs were Pgp+ and 8 (8/25, 32%) dogs were Pgp-. In the group of dogs treated with etoposide phosphate, 13 (13/23, 57%) dogs were Pgp+ and 8 (8/23, 35%) dogs were Pgp-P-glycoprotein expression could not be interpreted in 4 (4/48, 8%) dogs. There were no significant differences in Pgp tumor expression between the two treatment groups (*P* = 1.0). In the group of dogs previously treated with CHOP-based chemotherapy, 3 (3/7, 43%) dogs were Pgp+, 3 (3/7, 43%) were Pgp-, and one non evaluable.

### Safety analysis

All 48 dogs were evaluated for safety analysis during the induction chemotherapy protocol over a period of 8 weeks. Thirteen (13/25, 52%) dogs completed the 4-cycle induction protocol in the F14512 group and 15 (15/23, 65%) dogs in the etoposide phosphate group. Twelve (12/25, 48%) dogs discontinued the protocol in the F14512 group, 4 dogs due to progressive disease, and 8 dogs for death related to the disease. In the etoposide phosphate group, 8 (8/23, 35%) dogs did not complete the protocol, 4 for progressive disease and 4 dogs died due to the disease. Seventy-one cycles in the F14512 group and 73 cycles in the etoposide phosphate group were evaluated and the type, frequency and severity of treatment-emergent adverse events (TEAE) were graded according to the Veterinary Cooperative Oncology Group criteria for adverse events (VCOG-CTCAE), version 1.1 [27]. Treatment-emergent adverse events included neutropenia, anemia, thrombocytopenia, and gastrointestinal disorders (diarrhea and vomiting). No other biological adverse events were reported. Incidences of TEAEs are summarized in Table 2. Most TEAEs were moderate in intensity and all TEAEs were reversible. Hematologic adverse events were more important in number and severity in the F14512 group than in the etoposide phosphate group (*P* < 0.001). Neutropenia was observed 4 to 14 days (median: 8 days) after F14512 administration and 5 to 9 days (median: 8 days) after etoposide phosphate administration. Thrombocytopenia and anemia were reported 5 to 14 days after F14512 and etoposide phosphate administration. All thrombocytopenia and anemia events were asymptomatic and reversible. All non-hematological adverse events were gastrointestinal adverse events (diarrhea and vomiting) and were reported in 18 (18/25, 72%) dogs treated with F14512 and 22 (22/23, 96%) dogs treated with etoposide phosphate. Gastrointestinal adverse events were more frequent in the etoposide phosphate group compared with the F14512 group (*P* = 0.049). No hypersensitivity reactions were reported.

**Table 2:** Hematological and non-hematological adverse events graded according to the Veterinary Cooperative Oncology Group criteria for adverse events (VCOG-CTCAE), version 1.1

Adverse events	F14512 (71 cycles, 25 dogs)				Etoposide phosphate (73 cycles, 23 dogs)			
	Grade 1–2	Grade 3	Grade 4	Grade 5	Grade 1–2	Grade 3	Grade 4	Grade 5
**Number of cycles with hematological adverse events and febrile neutropenia, no. (%)**								
Neutropenia	19 (27%)	11 (15%)	27 (38%)	0 (0%)	20 (27%)	4 (5%)	2 (3%)	0 (0%)
Thrombocytopenia	16 (23%)	5 (7%)	4 (6%)	0 (0%)	12 (16%)	1 (1%)	0 (0%)	0 (0%)
Anemia	29 (41%)	3 (4%)	2 (3%)	0 (0%)	31 (42%)	0 (0%)	0 (0%)	0 (0%)
Febrile neutropenia	0 (0%)	1 (1%)	9 (13%)	0 (0%)	0 (0%)	0 (0%)	1 (1%)	0 (0%)
**Number of dogs with hematological adverse events and febrile neutropenia, no. (%)**								
Neutropenia	3 (12%)	5 (20%)	15 (60%)	0 (0%)	8 (35%)	4 (17%)	2 (9%)	0 (0%)
Thrombocytopenia	5 (20%)	4 (16%)	3 (12%)	0 (0%)	7 (30%)	1 (4%)	0 (0%)	0 (0%)
Anemia	15 (60%)	2 (8%)	2 (8%)	0 (0%)	16 (70%)	0 (0%)	0 (0%)	0 (0%)
Febrile neutropenia	0 (0%)	1 (4%)	9 (36%)	0 (0%)	0 (0%)	0 (0%)	1 (4%)	0 (0%)
**Number of dogs with non-hematological adverse events, no. (%)**								
Gastrointestinal adverse events	18 (72%)	0 (0%)	0 (0%)	0 (0%)	22 (96%)	0 (0%)	0 (0%)	0 (0%)
Vomiting	5 (20%)	0 (0%)	0 (0%)	0 (0%)	17 (74%)	0 (0%)	0 (0%)	0 (0%)
Diarrhea	17 (68%)	0 (0%)	0 (0%)	0 (0%)	21 (91%)	0 (0%)	0 (0%)	0 (0%)

All adverse events were manageable, reversible and resulted in prolonged or early hospitalization for 10 (10/25, 40%) dogs in the F14512 group (1 Grade 3 febrile neutropenia and 9 prolonged Grade 4 neutropenia) and for 1 (1/23, 4%) dog in the etoposide phosphate group (prolonged Grade 4 neutropenia). The median time of prolonged or early hospitalization was 3 days (range 111 days). Adverse events resulted in treatment dose reduction for 7 (7/25, 28%) dogs in the F14512 group and for 1 (1/23, 4%) dog in the etoposide phosphate group. No death related to treatment was reported.

Quality of life assessment, defined by a questionnaire completed by pet owners at day 0, 23 and 62, did not reveal any significant difference in quality of life between the two treatment groups (Supplementary Figure 1).

### Efficacy endpoints

All 48 randomized dogs started the treatment (Figure 1). Nineteen (19/25, 76%) dogs treated with F14512 achieved an objective response with 12 CR and 7 PR. In the etoposide phosphate group, 19 (19/23, 83%) dogs achieved an objective response with 9 CR and 10 PR. No significant difference was seen between the two groups (*P* = 0.73). The median time from randomization to best complete or partial response was very similar in the two groups: 26 days (range: 570 days) in F14512 group and 33 days (range: 565) in etoposide phosphate group (*P* = 0.81).

At day 62, at the end of 4 cycles, the remission status was assessed based on CT-imaging according to the Response Evaluation Criteria in Solid Tumors version 1.0 (RECIST) [28]. The overall response rate (day 62) was 44% with 7 (7/25, 28%) dogs experiencing a CR and 4 (4/25, 16%) dogs experiencing a PR in the F14512 group *versus* 48% with 7 (7/23, 31%) dogs experiencing a CR and 4 (4/23, 17%) dogs experiencing a PR in the etoposide phosphate group. There was no difference in overall response rate at day 62 between the two groups (*P* = 0.79).

Disease progression or death related to the disease was reported in all dogs. The median PFS for all dogs was 86 days (range: 4224). The median PFS for dogs treated with F14512 was 86 days (range: 7224), and 91 days (range: 4202) for those treated with etoposide phosphate, with no statistical difference between the two groups (*P* = 0.30). Five dogs were still alive at the time of data analysis (3 dogs in F14512 group and 2 dogs in etoposide phosphate group) with a median time of follow-up of 139 days (range: 84–153).

**Figure 3:**
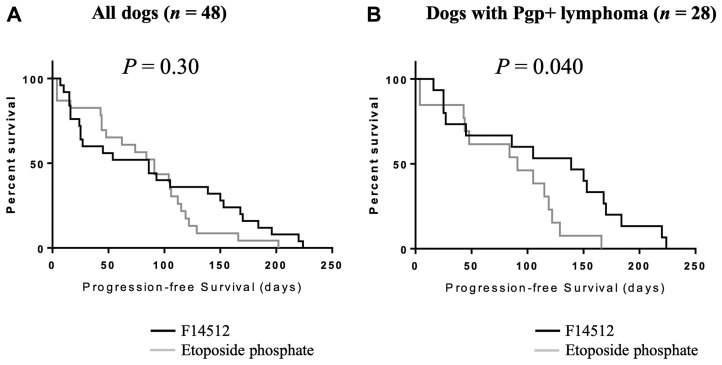
Kaplan-Meier curves by treatment group for progression-free survival. (**A**) The entire cohort, *n* = 48. (**B**) Dogs with Pgp-overexpressing lymphoma (Pgp+), *n* = 28. Log-rank *P* values are shown

Analysis of the 28 randomly assigned dogs with Pgp-overexpressing lymphoma (Pgp+), 10 (10/15, 67%) dogs treated with F14512 achieved an objective response (6 CR and 4 PR) at day 62 compared to 5 (5/13, 38%) dogs treated with etoposide phosphate (3 CR and 2 PR). Median PFS across treatments for dogs with Pgp-overexpressing lymphoma (Pgp+) was 105 days (range: 4–224). Median PFS for dogs with Pgp-overexpressing lymphoma (Pgp+) treated with F14512 was 139 days (range: 16–224) *versus* 91 days (range: 4–168) for dogs treated with etoposide phosphate. Kaplan-Meier curves for PFS are shown in Figure 3. Dogs with Pgp-overexpressing lymphoma (Pgp+) treated with F14512 were significantly more likely to have a longer PFS compared with dogs that received etoposide phosphate (HR: 0.42, 95%-CI: 0.17–0.99; *P* = 0.046).

### Exploratory biomarkers

#### Tumor cell survival and γ-H2AX expression

Formation of γ-H2AX has been identified as an early event after the production of double-strand breaks and has been widely used as a tool to measure induced DNA damage caused by cytotoxic chemical agents [29, 30]. We examined the possibility that expression of γ-H2AX after treatment initiation might be used as a surrogate indicator of DNA damage response.

Total surviving cells count and the proportion of γ-H2AX nuclear staining were measured by flow cytometry prior to treatment and following the ðrst injection of F14512 or etoposide phosphate to determine if treatment administration was associated with any cytotoxic effect. Serial fine-needle aspirates were performed in tumoral lymph nodes before treatment administration and two, four and 52 hours following the first drug injection. The total surviving cells count in these serial aspirates was evaluated using Trypan blue exclusion method and normalized to the aspirate volume.

**Figure 4:**
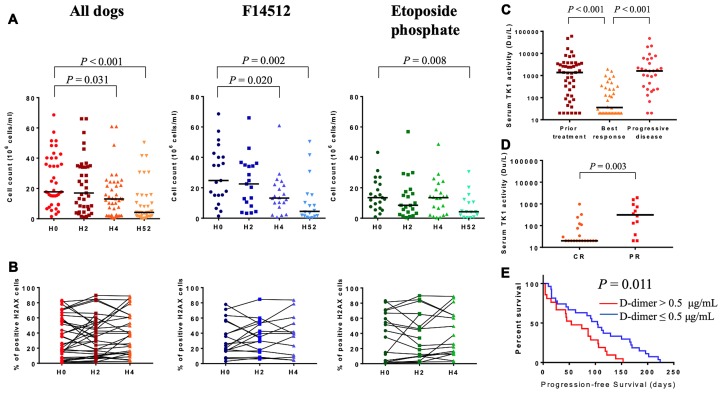
Exploratory biomarkers. (**A**) Total surviving cells count measured by flow cytometric analysis at H0, H2, H4 and H52 following treatment initiation. Drug infusion was started at H0. The horizontal bars represent median. (**B**) Percentage of γ-H2AX expression in surviving cells at H0, H2, H4 following treatment initiation. Groups were compared using Wilcoxon rank test. (**C**) Serum thymidine kinase 1 activity (Du/L) in dogs prior to treatment initiation (*n* = 48), at the time of the best clinical response (*n* = 36) and at the time of progressive disease (*n* = 30). Minimum detectable activity for this assay: 20 Du/L. The horizontal bars represent median. (**D**) Serum thymidine kinase 1 activity in dogs with complete (CR, *n* = 20) and partial (PR, *n* = 12) response. Minimum detectable activity for this assay: 20 Du/L. The horizontal bars represent median. Levels of sTK1 activity between groups were compared using Wilcoxon rank test. (**E**) Kaplan-Meier curve in dogs with high (> 0.5 μg/mL, *n* = 21) or low (≤ 0.5 μg/mL, *n* = 27) pretreatment D-dimer levels. Log-rank *P* value is shown.

A rapid and significant decrease in the number of total surviving cells was observed in the two treatment groups, as early as 4 hours after the initiation of the F14512 administration and after 52 hours after the beginning of etoposide phosphate administration (Figure 4A). Thirty-six dogs have pretreatment γ-H2AX nuclear expression interpretable (median: 30.73%, range: 0.59–83.16). Two (H2) and four hours (H4) after treatment initiation, surviving cells showed persistent elevated expression of γ-H2AX in both treatment groups (Figure 4B). A more than 10% increase of γ-H2AX nuclear positive surviving cells was observed in 11 dogs, as early as 4 hours after the beginning of F14512 and etoposide phosphate administration. In these 11 dogs, 8 (8/11, 73%) experienced a clinical response (5 CR, 3 PR). Among the non-responders with γ-H2AX nuclear expression interpretable (5 SD, 3 PD), static or decrease percentage of γ-H2AX nuclear positive surviving cells was observed in 6 (6/8, 62.5%) dogs 4 hours after the beginning of drug administration. No differences between F14512 and etoposide treatment groups were identified.

#### Serum thymidine kinase 1 activity (sTK1)

Serum thymidine kinase 1 (sTK1) activity has been shown to correlate with the proliferative activity of cancer cells providing information regarding prognosis and treatment effectiveness in human patients with hematologic malignancies [31–33]. To evaluate sTK1 activity as a potential blood biomarker of treatment efficacy, we measured sTK1 activity prior to treatment initiation, at the time of the best clinical response and at the time of relapse. Pretreatment sTK1 activity demonstrated a high degree of variation (median: 1374 Du/L, range: 20–60005, Supplementary Table 1). Pretreatment sTK1 activity was higher in dogs with B-cell lymphoma (*P* < 0.001), in dogs with clinical substage b (*P* = 0.025) and in dogs with Pgp-overexpressing lymphoma (*P* = 0.013). Pretreatment sTK1 activity was not correlated with the other clinical characteristics.

After treatment initiation, sTK1 activity decreased significantly when dogs experienced an objective tumor response (*P* < 0.001) and increased again at the time of relapse (*P* < 0.001) (Figure 4C). Serum TK1 activity was significantly lower in dogs with complete *versus* partial response (median: 20 vs. 312 Du/L, *P* = 0.003; Figure 4D).

#### Pretreatment plasma D-dimer levels

Here, we investigated the correlation between pretreatment plasma D-dimer levels and PFS. The median value of pretreatment plasma D-dimer concentration was 0.4 μg/mL (range: 0.1–14.3). The optimal cut-off value of D-dimer based on PFS was 0.5 μg/mL (HR: 2.218, 95%-CI: 1.175–4.187, *P* = 0.014). Pretreatment D-dimer levels > 0.5 μg/mL were not correlated with clinical characteristics (Supplementary Table 2). Median PFS for dogs with a pretreatment D-dimer level > 0.5 μg/mL was 54 days (range: 4–153) compared with 104 days (range: 10–224) for dogs with a D-dimer level ≤ 0.5 μg/mL. A pretreatment D-dimer level > 0.5 μg/mL was significantly associated with inferior PFS (*P* = 0.011, Figure 4E). In the multivariate Cox regression model, a D-dimer level > 0.5 μg/mL remained an independent predictor for worse PFS (HR: 3.197, 95%-CI: 1.536–6.655, *P* = 0.002, Table 3).

**Table 3:** Univariate and multivariate analysis of prognostic factors for progression-free survival

Variable	Univariate analysis		Multivariate analysis	
	HR (95%CI)	*P* value	HR (95%CI)	*P* value
Sex (male *vs*. female)	1.692 (0.943–3.038)	0.078		
Age (> 7 years *vs*. ≤ 7 years)	1.563 (0.862–2.837)	0.142		
Previous chemotherapy (yes *vs*. no)	2.711 (1.159–6.343)	0.021	8.371 (2.968- 23.613)	< 0.001
Stage (V *vs*. II+III+IV)	2.746 (1.267–5.954)	0.011	2.242 (0.982–5.119)	0.055
Systemic signs (b *vs*. a)	1.266 (0.711–2.257)	0.423		
Immunophenotype (T *vs*. B)	2.198 (0.931–5.190)	0.072		
Morphotype (DLBCL *vs*. others)	0.362 (0.185–0.709)	0.003	0.289 (0.142–0.586)	0.001
Pgp (Pgp+ *vs*. Pgp-)	0.631 (0.335–1.190)	0.155		
D-dimer level (> 0.5 *vs*. ≤ 0.5 μg/mL)	2.218 (1.175–4.187)	0.014	3.197 (1.536–6.655)	0.002
Treatment group (F14512 *vs*. Etoposide)	0.732 (0.403–1.332)	0.307		

DLBCL: Diffuse large B-cell lymphoma, Pgp: P-glycoprotein.

## DISCUSSION

The results of the present study provide evidence that naturally occurring cancer in dogs offers a unique opportunity for preclinical modeling to develop innovative therapies for human [3]. Here, we report the results of a randomized, double blind trial comparing the safety and antitumor activity of F14512 and etoposide phosphate in dogs with spontaneous NHL.

The administration of F14512 and etoposide phosphate demonstrated an acceptable safety profile in dogs and most adverse events were consistent with those expected with dose-intense chemotherapy. All TEAEs were reversible and no death related to treatment was reported. These results were consistent with the safety and tolerability data of F14512 and etoposide phosphate previously evaluated in dogs with lymphoma [25, 26] and revealed a similar safety proðle compared with clinical toxicology studies performed in humans. In the ðrst-in-man phase I trial of F14512 in adult human relapsed/refractory acute myeloid leukemia, hematologic toxicity was the major adverse event identified, including neutropenia (18%), febrile neutropenia (10%) and thrombocytopenia (5%) [De Botton S, Berthon C, Bulabois C, Prebet T, Vey N, Chevallier P. F14512 a novel polyamine-vectorized anti-cancer drug targeting topoisomerase II in adults patients with acute myeloid leukemia (AML): results from a Phase 1 study. Abstract presented at the 17th Congress of the European Hematology Association, Amsterdam, The Netherlands, 14 to 17 June 2012]. In human patients receiving etoposide, nausea and vomiting were the major gastrointestinal toxicities reported [34]. As in human patients, severe acute hypersensitivity reactions, caused by histamine release probably associated with the vehicle (polysorbate-80) used for the parenteral formulation, have been previously reported in dogs treated with etoposide (VP-16) [35, 36]. Etoposide phosphate is a water-soluble pro-drug of etoposide formulated without polysorbate-80 and no hypersensitivity reactions were reported in dogs during the protocol. This safety analysis demonstrates that clinical trials in dogs can provide an opportunity to assess toxicity data as a bridge to human therapeutics.

This study also confirmed the antitumor activity of F14512 and etoposide phosphate in spontaneous NHL in dogs. Both F14512 and etoposide phosphate induced an early decrease of tumoral lymph node cells and a high clinical response rate of 76% (19/25) and 83% (19/23) respectively. As a vectorized form of etoposide, F14512 had shown strong antitumor activity *in vitro* study [17] and superior therapeutic efficacy in xenograft mice models compared with etoposide [19, 20]. In our randomized double-blind trial, F14512 showed a comparable therapeutic efficacy to etoposide phosphate in term of response rate and PFS in the global treated population. This result might be explained by the better than expected outcome in etoposide phosphate group. Etoposide was previously shown to have a minimal therapeutic effect in a retrospective study on 13 dogs with naturally occurring lymphomas, with only 2 dogs (2/13, 15%) displaying a partial response [35]. We previously demonstrated that the IV formulation of etoposide phosphate improved the safety and tolerability of the drug in dogs with a maximum tolerated dose of 300 mg/m^2^ with daily IV infusion at 100 mg/m^2^, during 3 consecutive days every 2 weeks [26]. Here, we confirmed a valuable improvement of the therapeutic effect of etoposide phosphate for the treatment of lymphoma in dogs with an overall response rate of 83% compared with 15% in previous study. The observed clinical response of F14512 and etoposide phosphate reported here is comparable to the clinical response of doxorubicin, commonly recognized as the most efficient single-agent for the treatment of canine high-grade lymphoma, and associated with overall response rates reported from 74% to 84% [37–40]. Our study provides evidence that F14512 and etoposide phosphate demonstrate therapeutic potential in canine lymphoma, which should be further investigated either alone or in combination with other agents in dogs in this indication.

F14512 was also shown to be a poor substrate of the efflux protein Pgp. In the present study, the subgroup analysis of Pgp-overexpressing tumors showed a significant improvement in PFS in dogs with naturally Pgp-overexpressing NHL compared with etoposide phosphate. Progression-free survival was used as a primary endpoint because most of veterinary cancer patients do not die from their disease but are euthanized by their owners due to poor quality of life or financial limitations. This makes overall survival analysis much less robust than in human trials. This finding has important clinical implications. Indeed, following standard dose-intense chemotherapy, relapsed or refractory lymphomas develop chemotherapeutic multidrug resistance [10–12]. High expression of ABC-transports and P-glycoprotein in particular, has been associated with a decreased response to cytotoxic drugs, and a poor prognosis in human malignant hematopoietic tumors [15]. We evaluated the Pgp expression in naturally occurring tumor cells by IHC as previously described in canine lymphoma [7, 13, 41, 42] and the present study confirmed for the first time the superior antitumor activity of F14512 to etoposide phosphate in naturally occurring Pgp-overexpressing lymphomas in dogs. On the basis of these results, F14512 appears to be a potentially promising candidate for human clinical trials. F14512 has progressed to clinical development in human refractory/relapsing acute myeloid leukemia and ovarian cancer [43].

Biomarker studies, including γ-H2AX cytometry analysis, serum thymidine kinase 1 activity and pretreatment D-dimer level, revealed that spontaneous occurring lymphomas in dogs share clinical and biological similarities with their human counterparts, and provide opportunities to optimize drug development by generating relevant biomarker data unavailable with human xenografts or genetically engineered mice models [4–6]. Repeated analysis of γ-H2AX expression by flow cytometry was evaluated as a marker for DNA damage following treatment administration. In our study, the increase of γ-H2AX after drug administration was particularly higher in dogs who experienced a clinical response and the results confirmed that γ-H2AX can be useful for elucidating the pharmacodynamics of cytotoxic drugs and tumor targeting agents in dogs. Our study also demonstrated that sTK1 activity provides information regarding treatment response and disease progression, confirming previously results described in human NHL [31]. Monitoring sTK1 activity could help to detect complete responders and disease progression in dogs with lymphoma [44]. Pretreatment D-dimer levels have been reported to correlate with poor prognosis in several types of human malignancies, including DLBCL [45], however, the clinical significance of D-dimer has been rarely reported in dogs. This study demonstrated that high pretreatment D-dimer levels were independently associated with inferior PFS. D-dimer level, as a routinely used and easily measured marker, could serve as a useful prognostic predictor in clinical practice for both human and canine NHL.

Limitations of this study include the small number of dogs and the heterogeneity in morphological subtypes of canine lymphomas. However, randomized, double-blinded trial may overcome limitations of small sample size and yield valid conclusions [46, 47]. In the current study, the main clinical characteristics were balanced between the two treatment arms including sex, weight, stage, substage, immunophenotype, cytomorphological subtype, Pgp expression and prior treatment received. Dogs treated with F14512 were statistically younger than dogs treated with etoposide phosphate. However, no correlation was found between age and PFS and therefore, results in this present study would not be expected to have been significantly affected by the age of the dogs in the two treatment arms.

This study provided valuable preclinical evidence for the therapeutic potential of F14512 against NHL, however further clinical development in human resistant/refractory NHL would be warranted. Other molecular factors implicated in multidrug resistance and not evaluated in this study have been described in humans, including expression of breast cancer resistance proteins (BCRP), multidrug resistance-associated proteins 1 (MRP1), lung resistance proteins (LRP), p53 gene mutation and BCL-2 gene expression [10, 11, 15, 48–51].

In conclusion, the data reported here illustrate that spontaneous cancers in dogs offer a unique opportunity to integrate pet dog studies into the development paths of new cancer drugs. The final outcome of clinical trials in dogs would allow early assessment of drug activity and toxicity, and the identification and validation of biological endpoints and surrogate markers to provide significant rationale for future human clinical trials.

## MATERIALS AND METHODS

### Study design

The study design was prospective, randomized and double-blinded (dog’s owner-blind, investigator-blind) to compare the safety and the antitumor activity of F14512 with etoposide phosphate in dogs with NHL. The study design and the management of the data were carried out by OCR (Oncovet-Clinical-Research), Parc Eurasanté, Loos 59120, France. The clinical trial was conducted at Oncovet, Villeneuve d’Ascq 59650, France. F14512 was provided by Pierre Fabre Research Institute (Pierre Fabre Médicament, Toulouse 31100, France). The design and synthesis of F14512 have been patented (WO2005/100363). Etoposide phosphate, Etopophos®, was purchased from Bristol-Myers Squibb Pharmaceuticals Limited.

### Dog selection

The trial was available for client-owned dogs presenting with a histologically and/or cytologically confirmed diagnosis of intermediate to high-grade NHL. Dogs were considered eligible for inclusion if they (i) had new or previously diagnosed intermediate to high-grade NHL; (ii) had a measurable disease at the time of inclusion (allowing staging and clinical response assessment); (iii) had relapsed to standard therapies or whose owners had declined standard treatment; (iv) had no anticancer drug in the month before the inclusion (including steroids); (v) had no significant biochemical abnormality or blood cytopenia, which precluded the use of cytotoxic drugs; (vi) had no concurrent serious systemic disorder with an expected survival time of at least 9 weeks, according to the veterinary subjective assessment.

After enrolment, dogs were excluded of the trial in case of disease progression, severe adverse event, or withdrawal on owner or investigator decision, and a CHOP-based chemotherapy protocol was offered to the dog’s owner. Data collection was stopped after the exclusion or death of the last dog included in the study.

Ethical approval was obtained from the OCR (Oncovet-Clinical-Research) Ethical Committee before trial initiation. The protocol was performed in compliance with applicable local, national and international animal welfare regulations and adhered to the highest standards of animal care and use. Dogs were handled according to principles outlined in the National Institutes of Health (NIH) Guide for the Care and Use of Laboratory Animals. Written informed consent form was obtained from the owner before enrollment of each dog. Owners were permitted to withdraw their dog from the trial at any time.

### Randomization and masking

At enrollment, dogs were stratified regarding prior chemotherapy received (untreated *vs*. previously treated lymphoma) and randomly assigned to receive F14512 or etoposide phosphate in a ratio 1:1. It was planned to randomize 48 dogs. The randomization sequence was generated by an individual person who was independent of the study team by use of computer program. Investigators and dog’s owners were blinded to the treatment assignment.

### Procedures

#### Initial staging

At enrollment, all dogs were staged based on the modified WHO five-stage criteria for canine lymphoma. Initial staging tests included a complete blood count, biochemistry panel, whole-body CT-scan, peripheral lymph nodes fine-needle aspirates (FNA) and biopsies, ultrasound-guided liver and spleen FNA, urine analysis and a bone marrow aspirates. Bone marrow was considered infiltrated if neoplastic lymphoid cells represented ≥ 3% of all nucleated cells on the basis of bone marrow cytology [52]. Definitive diagnosis was confirmed for each dog with lymph node cytological examination by one board-certified clinical pathologist (C. Fournel-Fleury) and lymphoma was graded according to the updated Kiel morphological classification [53]. Immunophenotyping was performed using immunohistochemistry as previously described [25]. Cytology and immunophenotype were recorded for all dogs.

#### Treatment schedule

All the dogs involved in the study followed the same induction chemotherapy protocol over a period of 8 weeks (Figure 1). Dogs were randomly assigned to receive F145120.075 mg/kg, or etoposide phosphate100 mg/m^2^, delivered over a 3-hour intravenous (IV) infusion once daily on 3 consecutive days for 4 cycles, every 2 weeks (days 1–3, days 15–17, days 29–31, days 43–45). After completion of the 4 cycles, dogs who experienced a clinical benefit (complete or partial response or stable disease) received 3 additional consolidation cycles with one intravenous injection of the same drug every 3 weeks (day 68, 89, and 110).

The maximum tolerated dose of F14512 and etoposide phosphate was previously evaluated using two independent traditional 3+3 phase I dose-escalation trials [25, 26]. Dogs were hospitalized for 5 days during each cycle (days 1–5, days 15–19, days 29–33, and days 43–47). The prescribed dose was diluted in sterile saline solution for a total volume of 50 mL and was not discernable from the investigators and the healthcare team. The drug was administered IV through an indwelling catheter placed into a cephalic vein. No premedication was administered before the IV administration.

### Safety analysis

Safety analysis was evaluated in all dogs during the induction chemotherapy protocol over a period of 8 weeks. Safety evaluation were TEAEs, laboratory safety assessments and quality of life evaluation. Treatment-emergent adverse events were assessed at each scheduled visit and graded according to the VCOG-CTCAE, version 1.1 [27]. The adverse event grade assigned to each dog was based on the highest grade of hematologic toxicity reported, and the highest grade of gastrointestinal or constitutional signs noted. Owners were questioned about signs of adverse clinical effects using a quality of life questionnaire adapted from Lynch and colleagues and completed at day 0, 23 and 62 [54]. A complete blood count was performed before and 7 days after each cycle. Recovery of absolute neutrophil count to 1500 cells per μL and platelet count to 50 000 platelets per μL was required before starting each cycle. Dogs with prolonged (> 48 hours) asymptomatic grade 4 neutropenia, febrile neutropenia, and grade 3 gastrointestinal adverse events, the dose was reduced by 20%. If the neutrophils count was lower than 1500/μL or the platelets count was lower than 50 000/μL at the time of the next cycle, the chemotherapy administration was postponed for one week. If a delay of more than 2 weeks occurred, treatment was discontinued. A prophylactic broad-spectrum antibiotic therapy was administrated for dogs with grade ≥ 3 asymptomatic neutropenia. Dogs with febrile neutropenia, and grade ≥ 3 gastrointestinal adverse events were hospitalized and treated with intravenous fluids and antibiotics.

### Response assessment and follow-up

Response was evaluated before each chemotherapy administration. The best response to treatment was determined based on physical examination and peripheral lymph node size measurement using a caliper, according to the VCOG Response Evaluation Criteria for Peripheral Nodal Lymphoma in dogs version 1.0 [55]. At the end of 4 cycles (day 62), dogs underwent a complete end-staging and response to treatment were defined according to the RECIST, version 1.0 [28]. Bone marrow aspirations were repeated if infiltrated at enrollment.

A complete response (CR) was deðned as the disappearance of all measurable disease (i. e. lymph nodes returned to a size considered non-pathologic in the judgement of the evaluator and no new sites of disease should be observed). A partial response (PR) was deðned as at least 30% reduction in the sum of widest diameters of peripheral lymph nodes measured at enrollment. Stable disease (SD) was deðned after 4 cycles of treatment as <30% reduction or <20% increase in the sum of the widest diameters of the peripheral lymph nodes measured at enrollment. Progressive disease (PD) was deðned as >20% increase in the sum of the widest diameters of measurable peripheral lymph nodes or the appearance of new lesions. Progressive disease was confirmed with cytological evidence of lymphoma on lymph node aspirate.

Follow-up continued every 4 weeks from completion of treatment until disease progression, death or withdrawal from the study as a result of the owner’s decision.

### Immunohistochemistry for Pgp expression in tumor cells

P-glycoprotein expression in tumor cells was assessed using immunohistochemistry adapted from Lee and colleagues [13]. Experiments were performed on formalin-fixed paraffin-embedded samples. Immunohistochemical staining was performed automatically using with Ventana discovery XT using primary mouse anti-human P-glycoprotein Clone C494 antibody (ALX-801–003-C100, lab Enzo life sciences) and an HRP peroxydase secondary anti-mouse IgG antibody (lab Roche). Normal canine liver was used as a positive control for Pgp analysis. Non-immune serum substituted to C494 on lymph node tissues, was used as a negative control. All controls stained appropriately.

Immunohistochemical score (IHS) method was then evaluated by a scoring system adapted from Lee and colleagues described previously for canine lymphoma [13]. The scoring was based on the percentage of stained cells and the intensity of the staining. P-glycoprotein expression scoring was defined as follows: negative (less than 25% stained cells), low (more than 25% but less than 50% of the tumor cells are stained with a light staining), moderate (more than 50% but less than 75% of the tumor cells are stained with moderate intensity), high (more than 75% are stained with a moderate to high intensity). Samples were classified as Pgp-negative (Pgp-) when ≤ 50% of neoplastic cells in the field had cytoplasmic Pgp staining (negative and low); or as Pgp-positive (Pgp+) if >50% of neoplastic cells were positive (moderate and high). All slides were assessed by one board-certified veterinary pathologist (J. Hordeaux), who was blinded to the clinical information and treatment group of each dog.

### Exploratory biomarkers assessment

#### Gamma-H2AX cytometry analysis

Cells were collected via fine-needle aspirations from three different enlarged lymph nodes on which an average of three samples had been performed. Cells were collected before treatment initiation, and two, four and 52 hours following the first chemotherapy administration. Cell viability was measured using Nexcelom Cellometer Auto T4 (lab Ozyme Biosciences) and Covalab cell chambers as previously described [25]. Gamma-H2AX levels were quantified using the flow cytometry technique adapted from Huang and colleagues as previously described [25, 56]. Cells were ðxed in a 2% formalin solution and permeabilized in a 70% cold ethanol solution, then washed and rehydrated in PBS, 4% FCS and 0.1% Triton X-100. Nuclear H2AX staining was performed using a rabbit anti canine-gamma-H2AX antibody (NB100–384; lab Novus biologicals) and a secondary antibody (Cy5-goat anti-rabbit IgG A10931; lab Invitrogen Life Technologies).

#### DiviTumTM assay of serum Thymidine Kinase 1 (sTK1) activity measurement

Serum samples were collected for sTK1 activity measurement prior to treatment initiation, at the time of the best clinical response and at the time of progressive disease. Analysis of sTK1 activity was determined by a refined ELISA assay, the DiviTum^TM^ assay (lab Biovica International, Uppsala, Sweden), according to the manufacturer’s instructions (http://biovica.com/), as previously described [44]. The absorbance readings to DiviTum units per liter (Du/L) were converted using the values from standards with known TK activity, with a minimum detectable activity for this assay of 20 Du/L. Analyses were performed at the Biovica laboratory in Uppsala, Sweden, and investigators were blinded to dog and tumor data.

#### Pretreatment plasma D-dimers levels measurement

Plasma D-dimer concentrations were measured before treatment initiation using a turbidometric immunoassay (Nyco Card Reader II, NYCOMED) according to the manufacturer’s instructions.

### Study endpoints

The primary endpoints of the study were safety and progression-free survival (PFS). Safety analysis evaluated the occurrence of TEAEs graded according to the VCOG-CTCAE, version 1.1 [27]. Progression-free survival was measured from randomization to any of the following events, whichever occurred first: progression, relapse, death (related to the lymphoma or not), and evaluated according to the VCOG criteria v1.0 [55] or RECIST criteria v1.0 [28]. Secondary endpoints were overall response rate and time to response. Overall response rate was defined as the proportion of dogs with complete or partial response per VCOG or RECIST criteria and time to response was defined as the time from randomization to best clinical response. The exploratory endpoints were the analysis of nuclear expression of γ-H2AX, sTK1 activity and pretreatment plasma D-dimer level, as biomarker for treatment efficacy and prognosis in response to F14512 and etoposide phosphate administration.

### Statistical analysis

Categorical variables were expressed as numbers and percentages, and groups were compared with the Chi-square test or, for small numbers, Fisher’s exact test. Continuous normally distributed variables, data was expressed as mean and SD, and groups were compared with t-test or analysis of variance as appropriate. Non-normally distributed continuous variables were expressed as median and range, and groups were compared using Wilcoxon rank test or Krustal-Wallis test as appropriate. Time to response and PFS data were calculated using the Kaplan-Meier method and compared using the log-rank test. The Cox proportional hazard model was used for a univariate screen of all potential predictors of PFS. Variables with statistical and clinical significance were included in the multivariate analysis using a stepwise forward Cox regression model. Results were considered statistically significant with a two-sided *P* value < 0.05. The statistical analysis was performed using SPSS version 24.0 software (SPSS A, Inc., Chicago, IL, USA).

## SUPPLEMENTARY MATERIALS





## Abbreviations

ABC: ATP binding cassette
CR: complete response
CHOP: cyclophosphamide-doxorubicin-vincristine-prednisolone
DLBCL: diffuse large B-cell lymphoma
ELISA: enzyme-linked immunosorbent assay
FNA: fine-needle aspiration
IHC: immunohistochemistry
IHS: immunohistochemical score
MDR: multidrug resistance
(c)NHL: (canine) non-Hodgkin lymphoma
PD: progressive disease
PFS: progression-free survival
Pgp: P-glycoprotein
PR: partial response
PTS: polyamine transport system
RECIST: response evaluation criteria in solid tumors
SD: stable disease
TEAEs: treatment-emergent adverse events
(s)TK1: (serum) thymidine kinase 1
VCOG: Veterinary Cooperative Oncology Group
WHO: World Health Organization

## References

[1] Vail DM, MacEwen EG Spontaneously occurring tumors of companion animals as models for human cancer. Cancer Invest. 2000; 18:781–792. 10.3109/07357900009012210. 11107448

[2] Rowell JL, McCarthy DO, Alvarez CE Dog models of naturally occurring cancer. Trends Mol Med. 2011; 17:380–388. 10.1016/j.molmed.2011.02.004. 21439907 PMC3130881

[3] LeBlanc AK, Breen M, Choyke P, Dewhirst M, Fan TM, Gustafson DL, Helman LJ, Kastan MB, Knapp DW, Levin WJ, London C, Mason N, Mazcko C, et al Perspectives from man’s best friend: National Academy of Medicine’s Workshop on Comparative Oncology. Sci Transl Med. 2016; 8:324ps5. 10.1126/scitranslmed.aaf0746. 26843188 PMC7780241

[4] Ponce F, Marchal T, Magnol JP, Turinelli V, Ledieu D, Bonnefont C, Pastor M, Delignette ML, Fournel-Fleury C A morphological study of 608 cases of canine malignant lymphoma in France with a focus on comparative similarities between canine and human lymphoma morphology. Vet Pathol. 2010; 47:414–433. 10.1177/0300985810363902. 20472804

[5] Seelig DM, Avery AC, Ehrhart EJ, Linden MA The Comparative Diagnostic Features of Canine and Human Lymphoma. Vet Sci. 2016; 3:11. 10.3390/vetsci3020011. 28435836 PMC5397114

[6] Ito D, Frantz AM, Modiano JF Canine lymphoma as a comparative model for human non-Hodgkin lymphoma: recent progress and applications. Vet Immunol Immunopathol. 2014; 159:192–201. 10.1016/j.vetimm.2014.02.016. 24642290 PMC4994713

[7] Vajdovich P, Koltai Z, Dékay V, Kungl K, Harnos A Evaluation of Pgp (MDR1) immunohistochemistry in canine lymphoma - prognostic and clinical aspects. Acta Vet Hung. 2018; 66:309–328. 10.1556/004.2018.028. 29958524

[8] Tomiyasu H, Tsujimoto H Comparative Aspects of Molecular Mechanisms of Drug Resistance through ABC Transporters and Other Related Molecules in Canine Lymphoma. Vet Sci. 2015; 2:185–205. 10.3390/vetsci2030185. 29061940 PMC5644633

[9] Kourti M, Vavatsi N, Gombakis N, Sidi V, Tzimagiorgis G, Papageorgiou T, Koliouskas D, Athanassiadou F Expression of multidrug resistance 1 (MDR1), multidrug resistance-related protein 1 (MRP1), lung resistance protein (LRP), and breast cancer resistance protein (BCRP) genes and clinical outcome in childhood acute lymphoblastic leukemia. Int J Hematol. 2007; 86:166–173. 10.1532/IJH97.E0624. 17875533

[10] Gottesman MM, Fojo T, Bates SE Multidrug resistance in cancer: role of ATP-dependent transporters. Nat Rev Cancer. 2002; 2:48–58. 10.1038/nrc706. 11902585

[11] Kathawala RJ, Gupta P, Ashby CR, Chen ZS The modulation of ABC transporter-mediated multidrug resistance in cancer: a review of the past decade. Drug Resist Updat. 2015; 18:1–17. 10.1016/j.drup.2014.11.002. 25554624

[12] Mohammad IS, He W, Yin L Understanding of human ATP binding cassette superfamily and novel multidrug resistance modulators to overcome MDR. Biomed Pharmacother. 2018; 100:335–348. 10.1016/j.biopha.2018.02.038. 29453043

[13] Lee JJ, Hughes CS, Fine RL, Page RL P-glycoprotein expression in canine lymphoma: a relevant, intermediate model of multidrug resistance. Cancer. 1996; 77:1892–1898. 10.1002/(SICI)1097-0142(19960501)77:9<1892::AID-CNCR20>3.0.CO;2-U. 8646690

[14] Wuchter C, Leonid K, Ruppert V, Schrappe M, Büchner T, Schoch C, Haferlach T, Harbott J, Ratei R, Dörken B, Ludwig WD Clinical significance of P-glycoprotein expression and function for response to induction chemotherapy, relapse rate and overall survival in acute leukemia. Haematologica. 2000; 85:711–721. 10.1038/sj.leu.2401605. 10897123

[15] Damiani D, Michelutti A, Michieli M, Masolini P, Stocchi R, Geromin A, Ermacora A, Russo D, Fanin R, Baccarani M P-glycoprotein, lung resistance-related protein and multidrug resistance-associated protein in de novo adult acute lymphoblastic leukaemia. Br J Haematol. 2002; 116:519–527. 10.1046/j.0007-1048.2001.03322.x. 11849207

[16] Zandvliet M, Teske E, Schrickx JA Multi-drug resistance in a canine lymphoid cell line due to increased P-glycoprotein expression, a potential model for drug-resistant canine lymphoma. Toxicol *In Vitro*. 2014; 28:1498–1506. 10.1016/j.tiv.2014.06.004. 24975508

[17] Barret JM, Kruczynski A, Vispé S, Annereau JP, Brel V, Guminski Y, Delcros JG, Lansiaux A, Guilbaud N, Imbert T, Bailly C F14512, a potent antitumor agent targeting topoisomerase II vectored into cancer cells via the polyamine transport system. Cancer Res. 2008; 68:9845–9853. 10.1158/0008-5472.CAN-08-2748. 19047165

[18] Gentry AC, Pitts SL, Jablonsky MJ, Bailly C, Graves DE, Osheroff N Interactions between the etoposide derivative F14512 and human type II topoisomerases: implications for the C4 spermine moiety in promoting enzyme-mediated DNA cleavage. Biochemistry. 2011; 50:3240–3249. 10.1021/bi200094z. 21413765 PMC3086367

[19] Kruczynski A, Vandenberghe I, Pillon A, Pesnel S, Goetsch L, Barret JM, Guminski Y, Le Pape A, Imbert T, Bailly C, Guillaud N Preclinical activity of F14512, designed to target tumors expressing an active polyamine transport system. Invest New Drugs. 2011; 29:9–21. 10.1007/s10637-009-9328-3. 19777159

[20] Kruczynski A, Pillon A, Créancier L, Vandenberghe I, Gomes B, Brel V, Fournier E, Annereau JP, Currie E, Guminski Y, Bonnet D, Bailly C, Guilbaud N F14512, a polyamine-vectorized anti-cancer drug, currently in clinical trials exhibits a marked preclinical anti-leukemic activity. Leukemia. 2013; 27:2139–2148. 10.1038/leu.2013.108. 23568148

[21] Mouawad F, Gros A, Rysman B, Bal-Mahieu C, Bertheau C, Horn S, Sarrazin T, Lartigau E, Chevalier D, Bailly C, Lansiaux A, Meignan S The antitumor drug F14512 enhances cisplatin and ionizing radiation effects in head and neck squamous carcinoma cell lines. Oral Oncol. 2014; 50:113–119. 10.1016/j.oraloncology.2013.11.003. 24290982

[22] Brel V, Annereau JP, Vispé S, Kruczynski A, Bailly C, Guilbaud N Cytotoxicity and cell death mechanisms induced by the polyamine-vectorized anti-cancer drug F14512 targeting topoisomerase II. Biochem Pharmacol. 2011; 82:1843–1852. 10.1016/j.bcp.2011.08.028. 21924246

[23] Ballot C, Jendoubi M, Kluza J, Jonneaux A, Laine W, Formstecher P, Bailly C, Marchetti P Regulation by survivin of cancer cell death induced by F14512, a polyamine-containing inhibitor of DNA topoisomerase II. Apoptosis. 2012; 17:364–376. 10.1007/s10495-011-0681-2. 22127645

[24] Thibault B, Clement E, Zorza G, Meignan S, Delord JP, Couderc B, Bailly C, Narducci F, Vandenberghe I, Kruczynski A, Guilbaud N, Ferré P, Annereau JP F14512, a polyamine-vectorized inhibitor of topoisomerase II, exhibits a marked anti-tumor activity in ovarian cancer. Cancer Lett. 2016; 370:10–18. 10.1016/j.canlet.2015.09.006. 26404751

[25] Tierny D, Serres F, Segaoula Z, Bemelmans I, Bouchaert E, Pétain A, Brel V, Couffin S, Marchal T, Nguyen L, Thuru X, Ferré P, Guilbaud N, et al Phase I Clinical Pharmacology Study of F14512, a New Polyamine-Vectorized Anticancer Drug, in Naturally Occurring Canine Lymphoma. Clin Cancer Res. 2015; 21:5314–5323. 10.1158/1078-0432.CCR-14-3174. 26169968

[26] Boyé P, Serres F, Marescaux L, Hordeaux J, Bouchaert E, Gomes B, Tierny D Dose escalation study to evaluate safety, tolerability and efficacy of intravenous etoposide phosphate administration in 27 dogs with multicentric lymphoma. PLoS One. 2017; 12:e0177486. 10.1371/journal.pone.0177486. 28505195 PMC5432161

[27] Veterinary cooperative oncology group - common terminology criteria for adverse events (VCOG-CTCAE) following chemotherapy or biological antineoplastic therapy in dogs and cats v1.1. Vet Comp Oncol. 2016; 14:417–446. 10.1111/vco.283. 28530307

[28] Nguyen SM, Thamm DH, Vail DM, London CA Response evaluation criteria for solid tumours in dogs (v1.0): a Veterinary Cooperative Oncology Group (VCOG) consensus document. Vet Comp Oncol. 2015; 13:176–183. 10.1111/vco.12032. 23534501

[29] Martin OA, Bonner WM γ-H2AX in Cancer Cells: A Potential Biomarker for Cancer Diagnostics, Prediction and Recurrence. Cell Cycle. 2006; 5:2909–2913. 10.4161/cc.5.24.3569. 17172873

[30] Kuo LJ, Yang LX Gamma-H2AX - a novel biomarker for DNA double-strand breaks. *In Vivo*. 2008; 22:305–309. 18610740

[31] Hallek M, Wanders L, Strohmeyer S, Emmerich B Thymidine kinase: a tumor marker with prognostic value for non-Hodgkin’s lymphoma and a broad range of potential clinical applications. Ann Hematol. 1992; 65:1–5. 10.1007/BF01715117. 1643153

[32] Hallek M, Langenmayer I, Nerl C, Knauf W, Dietzfelbinger H, Adorf D, Ostwald M, Busch R, Kuhn-Hallek I, Thiel E, Emmerich B Elevated serum thymidine kinase levels identify a subgroup at high risk of disease progression in early, nonsmoldering chronic lymphocytic leukemia. Blood. 1999; 93:1732–1737. 10.1046/j.1365-2141.1996.d01-1796.x. 10029603

[33] O’Neill KL, Buckwalter MR, Murray BK Thymidine kinase: diagnostic and prognostic potential. Expert Rev Mol Diagn. 2001; 1:428–433. 10.1586/14737159.1.4.428. 11901857

[34] Sinkule JA Etoposide: a semisynthetic epipodophyllotoxin. Chemistry, pharmacology, pharmacokinetics, adverse effects and use as an antineoplastic agent. Pharmacotherapy. 1984; 4:61–73. 10.1002/j.1875-9114.1984.tb03318.x. 6326063

[35] Hohenhaus AE, Matus RE Etoposide (VP-16). Retrospective analysis of treatment in 13 dogs with lymphoma. J Vet Intern Med. 1990; 4:239–441. 10.1111/j.1939-1676.1990.tb03115.x. 2262925

[36] Siderov J, Prasad P, De Boer R, Desai J Safe administration of etoposide phosphate after hypersensitivity reaction to intravenous etoposide. Br J Cancer. 2002; 86:12–13. 10.1038/sj.bjc.6600003. 11857004 PMC2746527

[37] Mutsaers AJ, Glickman NW, DeNicola DB, Widmer WR, Bonney PL, Hahn KA, Knapp DW Evaluation of treatment with doxorubicin and piroxicam or doxorubicin alone for multicentric lymphoma in dogs. J Am Vet Med Assoc. 2002; 220:1813–1817. 10.2460/javma.2002.220.1813. 12092954

[38] Lori JC, Stein TJ, Thamm DH Doxorubicin and cyclophosphamide for the treatment of canine lymphoma: a randomized, placebo-controlled study. Vet Comp Oncol. 2010; 8:188–195. 10.1111/j.1476-5829.2010.00215.x. 20691026 PMC3129606

[39] Higginbotham ML, McCaw DL, Roush JK, Nietfeld JC, Wilkerson MJ, Reeds K, Burr D Intermittent single-agent doxorubicin for the treatment of canine B-cell lymphoma. J Am Anim Hosp Assoc. 2013; 49:357–362. 10.5326/JAAHA-MS-5929. 24051255

[40] Al-Nadaf S, Rebhun RB, Curran KM, Venable RO, Skorupski KA, Willcox JL, Burton JH Retrospective analysis of doxorubicin and prednisone as first-line therapy for canine B-cell lymphoma. BMC Vet Res. 2018; 14:356. 10.1186/s12917-018-1688-5. 30458771 PMC6245930

[41] Dhaliwal RS, Kitchell BE, Ehrhart E, Valli VE, Dervisis NG Clinicopathologic significance of histologic grade, pgp, and p53 expression in canine lymphoma. J Am Anim Hosp Assoc. 2013; 49:175–184. 10.5326/JAAHA-MS-5843. 23535752

[42] Sokołowska J, Urbańska K, Giziński S, Zabielska K, Lechowski R Immunohistochemical detection of P-glycoprotein in various subtypes of canine lymphomas. Pol J Vet Sci. 2015; 18:123–130. 10.1515/pjvs-2015-0016. 25928919

[43] Leary A, Le Tourneau C, Varga A, Sablin MP, Gomez-Roca C, Guilbaud N, Petain A, Pavlyuk M, Delord JP Phase I dose-escalation study of F14512, a polyamine-vectorized topoisomerase II inhibitor, in patients with platinum-refractory or resistant ovarian cancer. Invest New Drugs. 2019; 37:693–701. 10.1007/s10637-018-0688-4. 30547316 PMC6647401

[44] Boyé P, Floch F, Serres F, Geeraert K, Clerson P, Siomboing X, Bergqvist M, Sack G, Tierny D Evaluation of serum thymidine kinase 1 activity as a biomarker for treatment effectiveness and prediction of relapse in dogs with non-Hodgkin lymphoma. J Vet Intern Med. 2019; 33:1728–1739. 10.1111/jvim.15513. 31129922 PMC6639481

[45] Liu B, Li B, Zhou P, Yue W, Wang T, Wang J Prognostic value of pretreatment plasma D-dimer levels in patients with diffuse large B cell lymphoma (DLBCL). Clin Chim Acta. 2018; 482:191–198. 10.1016/j.cca.2018.04.013. 29649456

[46] Schulz KF, Chalmers I, Hayes RJ, Altman DG Empirical evidence of bias. Dimensions of methodological quality associated with estimates of treatment effects in controlled trials. JAMA. 1995; 273:408–412. 10.1001/jama.1995.03520290060030. 7823387

[47] Kjaergard LL, Villumsen J, Gluud C Reported methodologic quality and discrepancies between large and small randomized trials in meta-analyses. Ann Intern Med. 2001; 135:982–989. 10.7326/0003-4819-135-11-200112040-00010. 11730399

[48] Nuessler V, Stötzer O, Gullis E, Pelka-Fleischer R, Pogrebniak A, Gieseler F, Wilmanns W Bcl-2, bax and bcl-xL expression in human sensitive and resistant leukemia cell lines. Leukemia. 1999; 13:1864–1872. 10.1038/sj.leu.2401571. 10557064

[49] Filipits M, Jaeger U, Simonitsch I, Chizzali-Bonfadin C, Heinzl H, Pirker R Clinical relevance of the lung resistance protein in diffuse large B-cell lymphomas. Clin Cancer Res. 2000; 6:3417–3423. 10999723

[50] Suvannasankha A, Minderman H, O’Loughlin KL, Nakanishi T, Ford LA, Greco WR, Wetzler M, Ross DD, Baer MR Breast cancer resistance protein (BCRP/MXR/ABCG2) in adult acute lymphoblastic leukaemia: frequent expression and possible correlation with shorter disease-free survival. Br J Haematol. 2004; 127:392–398. 10.1111/j.1365-2141.2004.05211.x. 15521915

[51] Stengel A, Schnittger S, Weissmann S, Kuznia S, Kern W, Kohlmann A, Haferlach T, Haferlach C TP53 mutations occur in 15.7% of ALL and are associated with MYC-rearrangement, low hypodiploidy, and a poor prognosis. Blood. 2014; 124:251–258. 10.1182/blood-2014-02-558833. 24829203

[52] Marconato L, Martini V, Aresu L, Sampaolo M, Valentini F, Rinaldi V, Comazzi S Assessment of bone marrow infiltration diagnosed by flow cytometry in canine large B cell lymphoma: prognostic significance and proposal of a cut-off value. Vet J. 2013; 197:776–781. 10.1016/j.tvjl.2013.05.003. 23735731

[53] Fournel-Fleury C, Magnol JP, Bricaire P, Marchal T, Chabanne L, Delverdier A, Bryon PA, Felman P Cytohistological and immunological classification of canine malignant lymphomas: comparison with human non-Hodgkin’s lymphomas. J Comp Pathol. 1997; 117:35–59. 10.1016/S0021-9975(97)80065-5. 9263843

[54] Lynch S, Savary-Bataille K, Leeuw B, Argyle DJ Development of a questionnaire assessing health-related quality-of-life in dogs and cats with cancer. Vet Comp Oncol. 2011; 9:172–182. 10.1111/j.1476-5829.2010.00244.x. 21848620

[55] Vail DM, Michels GM, Khanna C, Selting KA, London CA, Veterinary Cooperative Oncology Group Response evaluation criteria for peripheral nodal lymphoma in dogs (v1.0)- a Veterinary Cooperative Oncology Group (VCOG) consensus document. Vet Comp Oncol. 2010; 8:28–37. 10.1111/j.1476-5829.2009.00200.x. 20230579

[56] Huang X, Darzynkiewicz Z Cytometric assessment of histone H2AX phosphorylation: a reporter of DNA damage. Methods Mol Biol. 2006; 314:73–80. 10.1385/1-59259-973-7:073. 16673875 PMC1458374

