# Supplementary material for: Randomized, double-blind trial of F14512, a polyamine-vectorized anticancer drug, compared with etoposide phosphate, in dogs with naturally occurring lymphoma
Source: Oncotarget. 2020 Feb 18;11(7):671–86. doi: 10.18632/oncotarget.27461 (PMC7041934; doi:10.18632/oncotarget.27461)
Supplement: Supplementary file 1 [file oncotarget-11-671-s001.pdf]

Randomized, double-blind trial of F14512, a polyamine-vectorized anticancer drug, compared with etoposide phosphate, in dogs with naturally occurring lymphoma

SUPPLEMENTARY MATERIALS

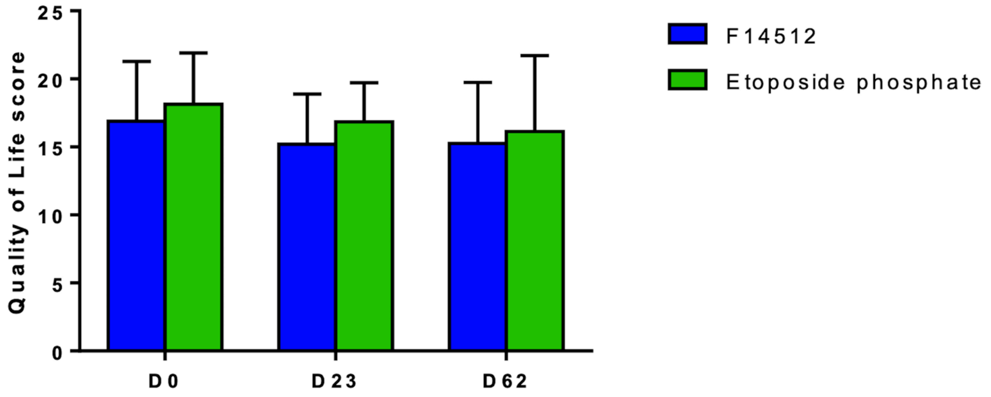

Supplementary Figure 1: Quality of life monitoring.

Supplementary Table 1: Serum thymidine kinase 1 activity measured prior to treatment according to clinical characteristics

Epidemiological characteristics	Number of dogs (%)	Pretreatment sTK1 activity, Du/L Median (range)	*P* value
**All population**	**48 (100%)**	1374 (20 – 60005)	
**Prior treatment**			0.15[a]
Untreated	**41 (85%)**	1638 (20 – 60005)	
Prior chemotherapy	**7 (15%)**	334 (20 – 7269)	
**Clinical stage**			0.12[b]
Stage II	**2 (4%)**	20 (20 – 20)	
Stage III	**8 (17%)**	1327 (20 – 3921)	
Stage IV	**29 (60%)**	1638 (20 – 60005)	
Stage V	**9 (19%)**	2994 (20 – 47453)	
**Clinical substage**			0.025[a]
Substage a	**26 (54%)**	934 (20 – 47453)	
Substage b	**22 (46%)**	3018 (20 – 60005)	
**Immunophenotype**			< 0.001[a]
B-cell lymphoma	**37 (77%)**	1688 (20 – 60005)	
T-cell lymphoma	**7 (15%)**	38 (20 – 834)	
**Cytomorphological Subtype**			0.18[a]
DLBCL	**33 (69%)**	1688 (20 – 60005)	
Others	**15 (31%)**	834 (20 – 47453)	
**Pgp**			0.013[a]
Pgp- (neg and low)	**16 (33%)**	280 (20 – 47453)	
Pgp+ (mid and high)	**28 (58%)**	2470 (20 – 60005)	
**Treatment group**			0.42[a]
F14512	**25 (52%)**	1309 (20 – 47453)	
Etoposide phosphate	**23 (48%)**	2551 (20 – 60005)	

DLBCL: Diffuse large B-cell lymphoma. Pgp: P-glycoprotein. (a) Wilcoxon rank test, (b) Kruskal-Wallis test.

Supplementary Table 2. The clinical characteristics and outcome of dogs with low (≤ 0.5 mg/mL, *n* = 27) or high (> 0.5 mg/mL, *n* = 21) pretreatment D-dimer levels

Parameters	Number of dogs (%)	Number of dogs (%) with D-dimer ≤ 0.5 μg/mL	Number of dogs (%) with D-dimer > 0.5 μg/mL	*P* value
**Overall**	**48 (100%)**	27 (100%)	21 (100%)	
**Clinical Stage**				0,15[a]
Stage II+III+IV	**39 (81%)**	24 (89%)	15 (71%)	
Stage V	**9 (19%)**	3 (11%)	6 (29%)	
**Clinical Substage**				0.24[a]
Substage a	**26 (54%)**	17 (63%)	9 (43%)	
Substage b	**22 (46%)**	10 (37%)	12 (57%)	
**Immunophenotype**				0.44[a]
B-cell	**37 (77%)**	22 (82%)	15 (71%)	
T-cell	**7 (15%)**	3 (11%)	4 (19%)	
**Cytomorphological Subtype**				0.76[a]
DLBCL	**33 (69%)**	18 (67%)	15 (71%)	
Others	**15 (31%)**	9 (33%)	6 (29%)	
**Prior treatment**				0.12[a]
Untreated	**41 (85%)**	21 (78%)	20 (95%)	
Prior chemotherapy	**7 (15%)**	6 (22%)	1 (5%)	
**Treatment group**				1.0[a]
F14512	**25 (52%)**	14 (52%)	11 (52%)	
Etoposide phosphate	**23 (48%)**	13 (48%)	10 (48%)	
**Pgp**				0.53[a]
Pgp- (neg and low)	**16 (33%)**	10 (37%)	6 (29%)	
Pgp+ (mid and high)	**28 (58%)**	14 (52%)	14 (67%)	
**Outcome**				
ORR at D62	**22 (46%)**	13 (48%)	9 (43%)	0.72[b]
PFS (range)	**86 (4-224)**	104 (10-224)	54 (4-153)	0.011[c]

DLBCL: Diffuse large B-cell lymphoma, ORR: overall response rate, PFS: progression-free survival, Pgp: P-glycoprotein. (a) Fisher's exact test; (b) Chi-square test; (c) Log-rank test.

Supplementary Table 3: Primary data. See Supplementary Table 3.
